# Injectable Thermosensitive Formulation Based on Polyurethane Hydrogel/Mesoporous Glasses for Sustained Co-Delivery of Functional Ions and Drugs

**DOI:** 10.3390/pharmaceutics11100501

**Published:** 2019-10-01

**Authors:** Monica Boffito, Carlotta Pontremoli, Sonia Fiorilli, Rossella Laurano, Gianluca Ciardelli, Chiara Vitale-Brovarone

**Affiliations:** 1 Department of Mechanical and Aerospace Engineering, Politecnico di Torino, Corso Duca degli Abruzzi 24, 10129 Torino, Italy; monica.boffito@polito.it (M.B.); rossella.laurano@polito.it (R.L.); gianluca.ciardelli@polito.it (G.C.); 2 Department of Applied Science and Technology, Politecnico di Torino, Corso Duca degli Abruzzi 24, 10129 Torino, Italy; carlotta.pontremoli@polito.it (C.P.); chiara.vitale@polito.it (C.V.-B.)

**Keywords:** polyurethane, injectable hydrogels, ion/drug delivery, mesoporous bioactive glasses, tissue regeneration

## Abstract

Mini-invasively injectable hydrogels are widely attracting interest as smart tools for the co-delivery of therapeutic agents targeting different aspects of tissue/organ healing (e.g., neo-angiogenesis, inflammation). In this work, copper-substituted bioactive mesoporous glasses (Cu-MBGs) were prepared as nano- and micro-particles and successfully loaded with ibuprofen through an incipient wetness method (loaded ibuprofen approx. 10% *w*/*w*). Injectable hybrid formulations were then developed by dispersing ibuprofen-loaded Cu-MBGs within thermosensitive hydrogels based on a custom-made amphiphilic polyurethane. This procedure showed almost no effects on the gelation potential (gelation at 37 °C within 3–5 min). Cu^2+^ and ibuprofen were co-released over time in a sustained manner with a significantly lower burst release compared to MBG particles alone (burst release reduction approx. 85% and 65% for ibuprofen and Cu^2+^, respectively). Additionally, released Cu^2+^ species triggered polyurethane chemical degradation, thus enabling a possible tuning of gel residence time at the pathological site. The overall results suggest that hybrid injectable thermosensitive gels could be successfully designed for the simultaneous localized co-delivery of multiple therapeutics.

## 1. Introduction

Tissue regeneration is the result of a complex process, which involves the synergistic contributes of cells, biomaterials, and bioactive factors (e.g., drugs, ions, growth factors). In most cases the complete regeneration of tissues fails due to a lack of vascularization; hence insufficient angiogenesis is one of the major current pathological concerns. In addition, the occurrence of drug-resistant infections may lead to several complications and greatly enhance the number of non-healing cases [1]. In order to overcome these issues, much attention has focused on the design of multifunctional systems which are able to simultaneously promote all the processes involved in the tissue/organ healing cascade. The incorporation of drugs, growth factors or inorganic nanomaterials into hydrogels has been extensively investigated [2,3], with the aim of combining in a single formulation several therapeutic abilities (such as pro-angiogenesis, anti-bacterial, anti-inflammatory). Among the approaches already explored, the combination of hydrogels with inorganic nanocarriers, which are able to incorporate and release several agents, represents a promising therapeutic strategy with a high potential for clinical translation. With this perspective, Zhu et al. prepared chitosan/mesoporous silica nanoparticle (MSN) composite hydrogels for localized co-delivery of drugs and macromolecules [4]. Similar hydrogel embedding ibuprofen-loaded MSN was proposed for surface coating of titanium implants [5] and poly(vinyl alcohol) hydrogels carrying oil-loaded MSNs containing curcumin were studied for dermal application [6]. More recently, a similar strategy has been explored to design thermosensitive hydrogels based on a poly(N-isopropylacrylamide) (PNIPAM) copolymer carrying mesoporous silica for dual-responsive (i.e., temperature and light) drug release [7] and for bone tissue regeneration [8]. 

More recently, in the field of tissue regeneration, considerable attention has been addressed towards the use of mesoporous bioactive glasses (MBGs) enriched with specific metallic ions (i.e., Sr^2+^, Cu^2+^, Ag^+^, Ce^3+^), as multifunctional therapeutic platforms for advanced medical devices [9,10]. Among the elements which can exert therapeutic properties, copper has been extensively investigated, due to the well documented pro-angiogenic effect alongside its antibacterial potential [11,12]. Several works have evidenced the antibacterial potential of Cu-containing MBGs against both planktonic bacteria and biofilms. In particular, Wu et al. [11] tested the successful antibacterial potential of Cu-containing MBG scaffolds against *Escherichia coli bacteria* and, accordingly with this, the authors recently demonstrated that Cu-containing MBG nanoparticles and their ionic extracts exert promising antibacterial activity against both Gram positive and Gram negative bacteria (*Staphylococcus aureus*, *Staphylococcus epidermidis* and *E. coli*) and are effective in inhibiting and preventing biofilm produced by *S. epidermidis* [13]. In addition to antibacterial abilities, Cu-substituted bioactive glasses were also demonstrated to stimulate angiogenesis and promote revascularization of soft and hard tissues [14,15].

Based on these promising results, we have recently reported the development of hybrid formulations able to combine the injectability of thermosensitive poly(ether urethane)-based (PEU) hydrogels and the capability of MBG particles to release functional copper ions [16]. This system was developed for a prolonged and sustained release of copper ions, with the final aim to promote simultaneously angiogenesis and anti-microbial effect [13,17,18,19]. However, no effects against persistent inflammation could be achieved with the formulated system. Hence, in order to expand the potentialities of the previously developed hybrid platform, Cu-substituted MBGs, in the form of micro- and nano-particles, were loaded with ibuprofen (Ibu), chosen as an anti-inflammatory agent, and embedded into PEU hydrogels. The resulting hybrid formulations were evaluated in terms of ion/drug co-delivery kinetics, gelation behavior and stability in an aqueous environment. 

Since the main goal of this contribution was the definition and the characterization of a novel and adaptable drug/ion release platform, our investigation mainly focused to identify the maximum amount of particles which could be incorporated within the hydrogel and to assess the related effect on the gelation behavior as well as the ion/drug co-release capacity that the final combined formulation could provide. The high potentiality of the proposed strategy lies in its wide versatility, as by tailoring the composition and cargo of MBGs, different ions and bioactive factors can be co-released according to the therapeutic effects required by the final targeted application. Moreover, their release timing and dosages can be finally optimized according to the requirements of each specific application, to make the released payload effective (i.e., releasing kinetics within the therapeutic window), while avoiding cytotoxic concentrations. In addition, the use of a custom-made PEU hydrogel as the vehicle phase of the MBGs provides the final system with a further degree of freedom. The characteristic LEGO-like chemical structure of PEUs allows polymer properties to be finely tuned by changing their constituting building blocks. This versatility opens the way to the design of stimuli-responsive hydrogels with an enhanced control over payload release and/or hydrogel dissolution/degradation, thus improving the release properties compared to those observed for MBGs alone. To the best of our knowledge, this is the first attempt to design a hybrid injectable formulation based on a thermo-sensitive hydrogel embedding MBGs able to co-release therapeutic ions and drugs.

## 2. Materials and Methods 

### 2.1. Synthesis of Cu-Substituted MBGs

#### 2.1.1. Materials

Cetyltrimethylammonium bromide (CTAB ≥ 98%), ammonium hydroxide solution (NH_4_OH), double distilled water (ddH_2_O), tetraethyl orthosilicate (TEOS), calcium nitrate tetrahydrate (Ca(NO_3_)_2_ ∙ 4H_2_O, 99%), copper chloride (CuCl_2_, 99%), Pluronic P123 (EO_20_PO_70_EO_20_, M_n_ ∼5800 Da), ibuprofen (>98% GC), Trizma^®^ base, primary standard and buffer, ≥99.9% (titration) were purchased from Sigma Aldrich, Milan, Italy and used as received. All solvents were purchased from Sigma Aldrich (Milan, Italy) in analytical grade.

#### 2.1.2. Cu-Substituted MBG Nanoparticles

Cu-substituted MBGs nanoparticles (nominal molar ratio Cu/Ca/Si = 2/13/85, hereafter named as MBG_Cu2%_SG) were prepared using a base-catalyzed template sol-gel synthesis, following an already optimized protocol [16]. In brief, 6.6 g of CTAB, acting as template, and 12 mL of NH_4_OH were dissolved in 600 mL of ddH_2_O under magnetic stirring (350 rpm) for 30 min. Then, 30 mL of TEOS, 4.888 g of Ca(NO_3_)_2 ∙_ 4H_2_O and 0.428 g of CuCl_2_ were added, and the obtained solution was vigorously stirred for 3 h at room temperature (RT). The powder was collected by centrifugation (Hermle Labortechnik Z326, Hermle LaborTechnik GmbH, Wehingen, Germany) at 10,000 rpm for 5 min, washed twice with ddH_2_O and once with absolute ethanol. The final precipitate was dried at 70 °C for 12 h and calcined at 600 °C in air for 5 h at a heating rate of 1 °C min^−1^ using a Carbolite 1300 CWF 15/5 (Carbolite Ltd., Hope Valley, UK), in order to completely remove CTAB.

#### 2.1.3. Cu-Substituted MBG Microspheres

Cu-substituted MBG microspheres (nominal molar ratio Cu/Ca/Si=2/13/85, hereafter named as MBG_Cu2%_SD) were synthesized following the aerosol-assisted spray-drying method reported by Pontremoli, Boffito et al. [16]. Briefly, 2.030 g of Pluronic P123, acting as template, were dissolved in 85 g of ddH_2_O. Simultaneously, in a separate batch, a solution of 10.73 g of TEOS was pre-hydrolyzed under acidic conditions using 5 g of an aqueous HCl solution at pH 2. The solution with TEOS was then added drop-wise into the template solution and kept stirring for half an hour. Then, 0.163 g of CuCl_2_ and 1.86 g of Ca(NO_3_)_2_ ∙ 4H_2_O were added. The final solution was stirred for 15 min and then sprayed with a Büchi, Mini Spray-Dryer B-290 (Büchi Labortechnik AG, Flawil, Switzerland), using N_2_ as atomizing gas (inlet temperature 220 °C, N_2_ pressure 60 mm Hg, feed rate 5 mL min^−1^). The obtained powder was finally calcined at 600 °C in air for 5 h at a heating rate of 1 °C min^−1^ using a Carbolite 1300 CWF 15/5 (Carbolite Ltd., Hope Valley, UK).

#### 2.1.4. Ibuprofen Loading Procedure

Ibuprofen was loaded into MBG_Cu2%_SG and MBG_Cu2%_SD through the incipient wetness method [20]. In brief, 0.1 g of both Cu-substituted MBGs were impregnated several times by dropping consecutive small aliquots of an ibuprofen solution in ethanol (at the final concentration of 30 mg/mL) onto the powders at RT. After each impregnation, ethanol was evaporated at 50 °C for 10 min and the dried powder mixed with a spatula. In order to completely fill the mesopores with ibuprofen the impregnation procedure was carried out with four 100 μL aliquots. Lastly, the obtained powders were dried at 50 °C overnight and named as follows: MBG_Cu2%_SG_Ibu and MBG_Cu2%_SD_Ibu.

### 2.2. Characterization of Cu-Substituted MBGs Loaded with Ibu

The morphology and particle size of the prepared powders were analyzed by field-emission scanning electron microscopy (FE-SEM) using a ZEISS MERLIN instrument (Oberkochen, Germany). For FE-SEM observations, 10 mg of MBG_Cu2%_SG_Ibu were dispersed in 10 mL of isopropanol using an ultrasonic bath (Digitec DT 103H, Bandelin, Berlin, Germany) for 5 min to obtain a stable suspension. A drop of the resulting suspension was put on a copper grid (3.05 mm Diam. 200 MESH, TAAB), allowed to dry and successively chromium-plated prior to imaging (Cr layer 7 nm). MBG_Cu2%_SD_Ibu sample was dispersed directly onto the double face carbon tape placed on a sample stub and then coated with a Cr layer. Compositional analysis of the powders was performed by energy dispersive spectroscopy (EDS; AZtec EDS, Oxford instruments, Abingdon-on-Thames, UK). EDS spectra were acquired on powder dispersed on carbon tape by analyzing an area of 75 × 50 μm^2^. Nitrogen adsorption–desorption isotherms were measured by using an adsorption analyzer ASAP2020 Micromeritics (ASAP 2020 Plus Physisorption, Norcross, GA, USA) at a temperature of –196 °C. Before nitrogen adsorption–desorption measurements, loaded samples were outgassed at 37 °C for 5 h, in order to avoid the degradation of the drug. The Brunauer–Emmett–Teller (BET) equation was used to calculate the specific surface area (SSA_BET_) from the adsorption data (relative pressures 0.04–0.2). The pore size distribution was calculated through the DFT method (density functional theory) using the NLDFT kernel of equilibrium isotherms (desorption branch). The mesopore structure was investigated by transmission electron microscopy (TEM, Fei Company, Hillsboro, OR, USA) using a FEI Tecnai G2 operated at 200 kV. The samples were prepared by suspending the powders in ethanol and drop-wise placed on carbon coated copper grids. Thermo-gravimetric analysis (TGA) of the samples was performed on a TG 209 F1 Libra instrument from Netzsch (Selb, Germany) over a temperature range of 25–600 °C under air flux at a heating rate of 10 °C min^−1^. The drug content was determined from the weight loss between 200 and 600 °C, by applying a correction for the weight loss in the same range of temperature due to the surface silanol condensation as recorded on Cu-substituted MBGs before Ibu loading. Fourier transformed infrared (FT-IR) spectra of the drug-loaded samples were collected on a Bruker Equinox 55 spectrometer (Bruker, Billerica, MA, USA) over a range of wavenumbers from 4000 to 400 cm^−1^ (resolution 2 cm^−1^). In order to assess the amorphous state of incorporated Ibu, X-ray patterns were collected using an X’Pert PRO, PANalytical instrument (X’Pert PRO, PANalytical, Almelo, The Netherlands) (CuKα radiation at 40 kV and 40 mA). Data were obtained from 10° to 80° (diffraction angle 2ϑ) at a step size of 0.0130° and a scan step time of 60 s. Differential scanning calorimetry (DSC) analysis of Ibu-loaded samples was carried out with a DSC 204 F1 Phoenix (Netzsch) instrument ((Selb, Germany). The samples were heated from 37 °C to 200 °C at a heating rate of 10 °C min^−1^ under N_2_ flux.

### 2.3. Synthesis of Amphiphilic Poly(ether urethane) 

#### 2.3.1. Materials

Amphiphilic water-soluble PEU was synthesized starting from Poloxamer® 407 (P407, poly(ethylene oxide)-poly(propylene oxide)-poly(ethylene oxide) PEO-PPO-PEO triblock copolymer, 70% PEO, M_n_ 12,600 g/mol), 1,6-hexamethylene diisocyanate (HDI) and 1,4-cyclohexanedimethanol (CDM), purchased from Sigma Aldrich, Milan, Italy. Before the synthesis, all the reagents were anhydrified according to the following protocols: P407 was dried for 8 h at 100 °C and then cooled down at 40 °C at a pressure lower than 200 mbar; HDI was distilled under reduced pressure; CDM was vacuum-dried in a dessicator at RT. Dibutyiltin dilaurate (DBTDL) was also purchased from Sigma Aldrich, Milan, Italy and used as received to catalyze the polymerization reaction. Anhydrous 1,2-dichoroethane (DCE, Carlo Erba Reagents, Cornaredo, Italy) was prepared by pouring the solvent over activated (at 120 °C, overnight) molecular sieves (4Å, Sigma Aldrich, Milano, Italy) under N_2_ atmosphere for 8 h. All other solvents were purchased from Carlo Erba Reagents, Cornaredo, Italy in the analytical grade and used as received.

#### 2.3.2. Poly(ether urethane) Synthesis

PEU synthesis was carried out according to Boffito et al. [21]. P407, HDI and CDM were reacted at 1:2:1 molar ratio and the synthesis was conducted under N_2_ atmosphere following a prepolymerization method with a first reaction between P407 and HDI to form an N=C=O-terminated prepolymer which was then chain extended through the addition of CDM. In detail, the required amount of HDI was added to a 20% *w*/*v* concentrated P407 solution previously prepared in DCE and equilibrated at 80 °C. DBTDL was also added at 0.1% *w*/*w* concentration with respect to P407 and the reagents reacted for 150 min. Then, the reaction mixture was cooled down at 60 °C and a 3% *w*/*v* concentrated CDM solution previously prepared in DCE was added to start the chain extension reaction which lasted 90 min and was finally terminated through the addition of MeOH. The synthesized PEU was collected by precipitation in an excess of petroleum ether (petroleum ether: DCE = 4:1 *v*/*v*) and further purified by solubilization in DCE (20% *w*/*v*) and precipitation in a diethyl ether/MeOH mixture (98:2 *v*/*v*, 5:1 volume ratio with respect to DCE). PEU was collected by centrifugation (MIKRO 220R, Hettich, Tuttlingen, Germany), dried overnight, grinded and stored under vacuum at 4 °C until use. Hereafter, the synthesized PEU will be referred to with the acronym CHP407, where C, H and P407 refer to CDM, HDI and Poloxamer® 407, respectively. 

### 2.4. Chemical Characterization of the As-Synthesized PEU

The successful synthesis of CHP407 poly(ether urethane) was assessed by attenuated total reflectance Fourier transformed infrared (ATR-FTIR) spectroscopy and size exclusion chromatography (SEC). CHP407 and P407 ATR-FTIR spectra were registered using a Perkin Elmer (Waltham, MA, USA) Spectrum 100 instrument equipped with an ATR diamond crystal (UATR KRS5). Spectra were obtained as an average of 16 scans registered at RT within the spectral range 4000–600 cm^−1^ (resolution 4 cm^−1^). CHP407 molecular weight distribution was assessed by SEC using an Agilent Technologies 1200 Series (Agilent Technologies, Inc., Santa Clara, CA, USA) instrument equipped with a refractive index detector (RID) and two columns (Waters Styragel HR1 and HR4, Waters Corporation, Sesto San Giovanni, Italy). Analyses were conducted at 55 °C using N,N-dimethylformammide (DMF, CHROMASOLV Plus, inhibitor-free, for HPLC, Carlo Erba Reagents, Cornaredo, Italy), added with 0.1% *w*/*v* LiBr (Sigma Aldrich, Milano, Italy) as eluent at a flow rate of 0.5 mL/min. Samples were prepared at 2 mg/mL concentration in the mobile phase and filtered through a 0.45 μm syringe filter (Macherey-Nagel, Düren, Germany poly(tetrafluoro ethylene) membrane) before analysis. The Agilent ChemStation software was finally used to estimate CHP407 Number Average Molecular Weight (M_n_) and polydispersity index (D) based on a calibration curve previously constructed starting from 10 poly(methyl methacrylate) standards with M_n_ ranging between 940 and 214,600 g/mol.

### 2.5. Preparation of CHP407-Based Hydrogels Carrying Cu-MBGs Loaded with Ibu

CHP407 thermosensitive hydrogels were prepared at a final PEU concentration of 15% *w*/*v* [16,21]. Cu-MBGs loaded with Ibu were encapsulated into CHP407-based hydrogels with final particle concentration of 20 mg/mL. In detail, hybrid hydrogels were prepared by adding an aliquot of particle suspension in ddH_2_O (100 mg/mL), previously sonicated for 3 min at 20% amplitude, to a CHP407-based solution prepared in physiological saline solution (0.9% NaCl). The starting concentration of CHP407 solution was determined in order to reach the desired PEU and particle contents in the final system. Both CHP407 solubilization and particle addition were carried out at 4 °C to avoid undesired gelation during sample preparation. Sample mixing was conducted using a Vortex mixer for 30 s to ensure homogeneous particle dispersion within the sol-gel systems. Particles were added to CHP407 aqueous solutions immediately before use to avoid premature payload release before characterization tests. Pure CHP407 sol-gel systems and CHP407 hydrogels loaded with Ibu were also prepared as control samples. Loading of Ibu in CHP407 sol-gel systems was carried out by adding CHP407 aqueous solutions prepared at higher concentration with a predefined volume of an Ibu stock solution (at 40 mg/mL in ethanol) to reach an average content equal to the Ibu amount incorporated into MBG_Cu2%_SG_Ibu and MBG_Cu2%_SD_Ibu, as assessed through TGA analysis. Based on this calculation, CHP407 sol-gel systems were loaded with Ibu at a final concentration of 2.5 mg/mL. Hereafter, the developed sol-gel systems will be referred to with the acronyms reported in Table 1. For all the conducted tests, hydrogels were prepared in Bijou sample containers (inner diameter 17 mm, Carlo Erba Reagents, Cornaredo, Italy) at a final volume of 1 mL, to avoid geometry and volume influence on the performed characterizations.

### 2.6. Characterization of Hybrid Sol-Gel Systems

The thermosensitive behavior of pure CHP407 (i.e., without particle) and hybrid hydrogels (i.e., containing MBG_Cu2%_SG_Ibu, MBG_Cu2%_SD_Ibu or Ibu) was studied through tube inverting test in temperature ramp mode and isothermal conditions (i.e., at 37 °C) to assess the effects of drug/particle incorporation on hydrogel sol-to-gel transition. In view of their potential clinical application as injectable depots for the prolonged and sustained payload release, the delivery profile of ibuprofen and copper ion was investigated in physiological mimicking conditions. Furthermore, gel swelling potential and residence time in watery environment at physiological temperature were also assessed.

#### 2.6.1. Thermosensitive Behavior of CHP407-Based Sol-Gel Systems

Tube inverting test in temperature ramp conditions was carried out to estimate hydrogel Lower Critical Gelation Temperature (LCGT). In detail, samples were subjected to a step-by-step temperature increase within the range 5–40 °C at a rate of 1 °C/step. In each step, temperature was kept constant for 5 min and the samples were finally inverted for 30 s to visually inspect their sol, biphasic or gel state (in the gel state no flow was observed within the set inversion time). The same test was carried out in isothermal conditions at 37 °C to assess hydrogel gelation time. In this test, instead of temperature, incubation time at 37 °C was varied from 1 to 10 min with a rate of 1 min/step. Each step consisted in sample equilibration in the sol state for 10 min, incubation at 37 °C for the required time, inversion for 30 s and visual inspection.

#### 2.6.2. Gel Swelling and Stability in Aqueous Media

Gel swelling and stability in physiological mimicking conditions, that is, in aqueous media and 37 °C, were investigated on the designed sol-gel systems up to 14 days incubation time [16]. Samples (1 mL) were first incubated at 37 °C (Memmert IF75, Schwabach, Germany) for 15 min to allow a complete gelation and then added with 1 mL of Trizma^®^ (0.1 M, pH 7.4) previously equilibrated at 37 °C. Complete medium refresh was performed every two days. On predefined time points of 6h, 1d, 3d, 7d and 14d three samples were collected, weighted upon removal of the residual buffer, lyophilized (Martin Christ ALPHA 2–4 LSC, Osterode am Harz, Germany) and weighted again. The collected data were finally used to estimate sample percentage of swelling (weight change in wet state %) and polymer weight loss (weight loss in dry state %) [16]. Samples collected on day 14 were also analyzed by SEC to assess changes in polymer molecular weight distribution during incubation in aqueous media at 37 °C.

#### 2.6.3. Payload Release Studies

Payload release studies were carried out on hydrogels incorporating MBG_Cu2%_SG_Ibu, MBG_Cu2%_SD_Ibu and simply dispersed Ibu. Tests were conducted at 37 °C up to 14 days using Trizma^®^ as release medium. Complete gelation of the samples was ensured through incubation at 37 °C for 15 min; then, 1 mL of release medium (previously equilibrated at 37 °C) was added to each sample and the release test started. Release media were collected and completely refreshed at 1h, 3h, 5h, 1d, 2d, 3d, 4d, 8d, 10d, and 14d incubation time. Released Ibu was quantified through a high performance liquid chromatography (HPLC, Thermo Scientific, Dionex Ultimate 3000, Waltham, MA, USA) instrument equipped with a C18 column (5 μm, 120 Å) according to the protocol described by Alsirawan et al. [22]. A mixture of acetonitrile (ACN, Carlo Erba Reagents, Cornaredo, Italy, HPLC grade) and phosphoric acid solution at 0.03% *w/v* concentration (pH 2.25) at 60/40 *v/v* was used as mobile phase at 1.7 mL/min. Analysis were conducted with an injection volume of 20 μL, at RT and 214 nm for 5 min. In order to prepare the samples, the collected extracts were mixed with ACN at 40/60 volume ratio and filtered through a 0.45 μm syringe filter (Macherey-Nagel, Düren, Germany, poly(tetrafluoro ethylene) membrane). Ibu content was finally quantified with respect to a calibration curve based on ibuprofen standards with concentration ranging between 0 and 2.5 mg/mL. The collected extracts were also characterized by inductively coupled plasma atomic emission spectrometry technique (ICP-AES) (ICP-MS, Thermo Scientific, Waltham, MA, USA) to measure the concentration of released copper ions. In order to express the results in terms of released percentage, the amount of copper initially incorporated into the MBG framework was measured by dissolving MBG_Cu2%_SG_Ibu and MBG_Cu2%_SD_Ibu in a mixture of nitric and hydrofluoric acids (0.5 mL of HNO_3_ and 2 mL of HF for 10 mg of powder) and quantifying copper concentration through ICP-AES. To compare the ion/drug release kinetics from MBGs as such and embedded within the hydrogel, release tests from particle alone were also conducted as follows: drug-loaded powders were dispersed in physiological saline solution at 20 mg/mL MBG concentration and Trizma^®^ was added as release medium at 37 °C (1:1 volume ratio), as previously performed with the hydrogel systems. Release tests from free MBG particles were conducted up to 24 h observation time. In order to better characterize copper ion and ibuprofen release mechanism from the hydrogels incorporating MBG_Cu2%_SG_Ibu, MBG_Cu2%_SD_Ibu and Ibu as such, the Korsmeyer-Peppas equation was used in the form recently reported by Boffito et al. [23] to estimate the release exponent *n*, whose value classifies the type of release. Specifically, an *n* value of 0.45 or 0.89 is typical of a diffusion- or swelling/relaxation-controlled release, respectively. An *n* value within the range 0.45–0.89 characterizes an anomalous release, meanwhile when *n* shows a value higher than 0.89 other processes are ongoing in addition to diffusion and swelling/relaxation.

### 2.7. Statistical Analysis

Statistical analysis was performed on the data collected from swelling, stability, and payload release tests. All tests were performed in triplicate and results have been reported as mean ± standard deviation. Comparison among results was performed through GraphPad Prism (GraphPad Software, Inc., La Jolla, CA, USA, version 5.03, 2009; http://www.graphpad.com) using a Two-way ANOVA analysis followed by Bofferoni’s multiple comparison tests. The degree of statistical difference among the results was defined in accordance to Boffito et al. [21].

## 3. Results and Discussion

### 3.1. Cu-MBGs Loaded with Ibu

#### Morphological and Structural Characterization

**Figure 1:**
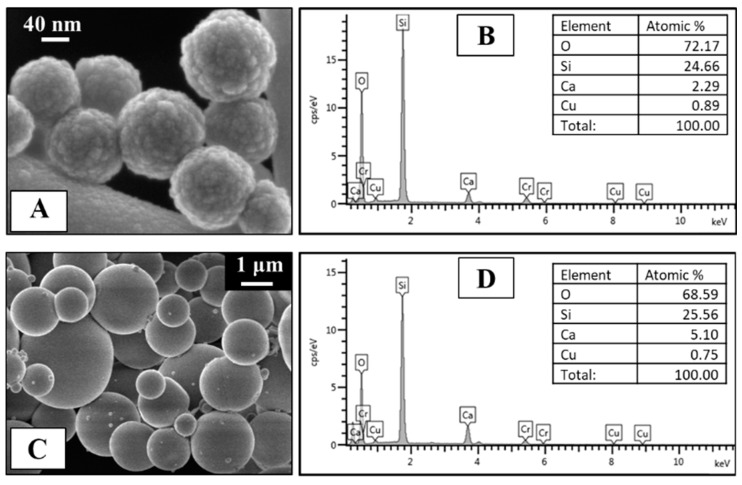
Field-emission scanning electron microscopy (FE-SEM) image of MBG_Cu2%_SG_Ibu (**A**) and MBG_Cu2%_SD_Ibu (**C**). Energy dispersive spectroscopy (EDS) spectrum of MBG_Cu2%_SG_Ibu (**B**) and MBG_Cu2%_SD_Ibu (**D**).

FE-SEM images of MBG_Cu2%_SG_Ibu (Figure 1A) and MBG_Cu2%_SD_Ibu (Figure 1C) showed nanoparticles with a monodispersed spherical shape (size range: 150–200 nm) and microspheres in the range of 1–5 μm, respectively. EDS spectra (Figure 1B,D) of both powders confirmed the presence of copper inside the framework, with a Cu/Si molar ratio in good agreement with the nominal ratio for both MBGs.

FESEM observations and EDS analysis evidenced that ibuprofen incorporation did not significantly alter the morphological features and the chemical composition of Cu-substituted MBGs, which resulted very similar to those reported for not-loaded samples [16]. In particular, the amount of copper revealed by EDS before and after the drug loading resulted unaffected, evidencing that the loading procedure did not induce any copper release (data not shown).

**Figure 2:**
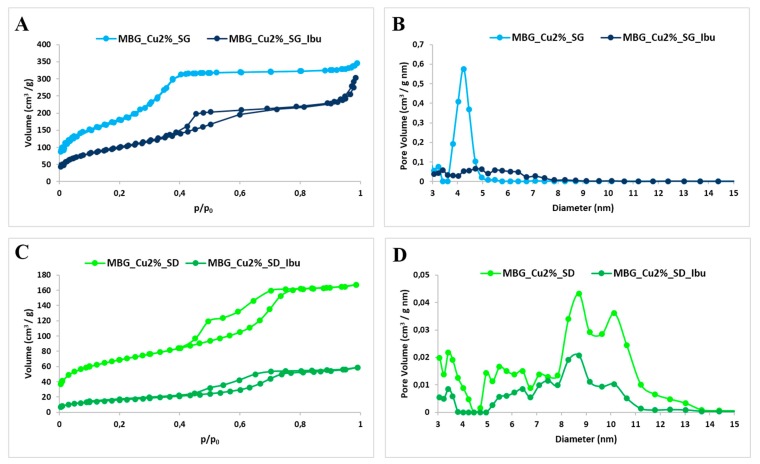
N_2_ adsorption–desorption isotherm of MBG_Cu2%_SG and MBG_Cu2%_SG_Ibu (**A**), MBG_Cu2%_SD and MBG_Cu2%_SD_Ibu (**C**). DFT (density functional theory) pore size distribution of MBG_Cu2%_SG and MBG_Cu2%_SG_Ibu (**B**), MBG_Cu2%_SD and MBG_Cu2%_SD_Ibu (**D**).

Figure 2 shows the nitrogen adsorption–desorption isotherms and the pore size distribution of the sample before and after drug loading. As expected, before loading, MBG_Cu2%_SG showed a type IV sorption isotherm, according to the IUPAC classification, with a well-defined step around 0.4 (P/P_0_), indicative of uniform mesopores. The specific surface area and pore volume values reported in Table 2 are characteristic of mesoporous materials with uniform pores and remarkable value of specific surface area (SSA_BET_) [24]. The mesopore size distribution was centered at around 4.2 nm, thus allowing the incorporation of ibuprofen whose molecular size is about 1 nm [25,26,27]. As expected, drug up-loading induced a significant modification of the adsorption-desorption isotherm (reduction of the adsorbed volume and presence of hysteresis loop) and a drastic reduction of the pore volume, as shown in Figure 2. In particular, the modification of the isotherm curve upon drug incorporation suggested that most of the mesopores were completely filled with Ibu. On the other hand, the remaining population underwent size reduction and shape modification from cylinder to ink-bottle pores, in analogy to the results reported by Hong et al. [28] for similar systems. In addition, the drastic reduction of pore volume as a consequence of drug incorporation was confirmed by the disappearance of the component centered at 4.2 nm (Figure 2B). The isotherm of MBG_Cu2%_SD was a type IV curve (Figure 2C), with H1 hysteresis loop, typical of mesoporous material with pores larger than 4 nm. Although the specific surface area was lower compared to that of MBG_Cu2%_SG, it resulted much higher compared to not-templated sol-gel glasses (few m^2^/g), conferring increased surface reactivity to MBGs in the biological environment [29]. The worm-like mesoporous structure was further confirmed by TEM images, reported in Figure S1. The pore size distribution evidenced multisized pores in the range between 8 and 11 nm (Figure 2D), which easily allows the diffusion and incorporation of ibuprofen molecules. A drastic reduction in SSA_BET_ was observed in MBG_Cu2%_SD_Ibu sample, while the pore volume reduction was lower compared to MBG_Cu2%_SG_Ibu sample (Table 2). Hence, in MBG_Cu2%_SD the incorporation of drug molecules occurred without a full occlusion of the available pore volume. The total amount of loaded drug was quantified by TGA analysis on both samples after drug incorporation. As reference, TGA analysis was also conducted on not-loaded MBG samples, proving the complete absence of residual organic species at 600 °C. TGA thermograms of MBG_Cu2%_SG_Ibu and MBG_Cu2%_SD_Ibu exhibited a significant weight decrease between 300 and 400 °C, which can be ascribed to ibuprofen loss [30], due to the rupture of the multiple H-bonding interactions between the drug and the hydroxyl groups of the inner MBG surface, in accordance with Mellaerts and co-workers [26]. The weight percentage of loaded ibuprofen, based on TGA analysis, turned out to be 12% in MBG_Cu2%_SG and 10% in MBG_Cu2%_SD. These results confirmed that the drug loading capacity increases with the increase of MGB surface area and pore volume, according to data reported in the literature for mesoporous silicas [31].

**Figure 3:**
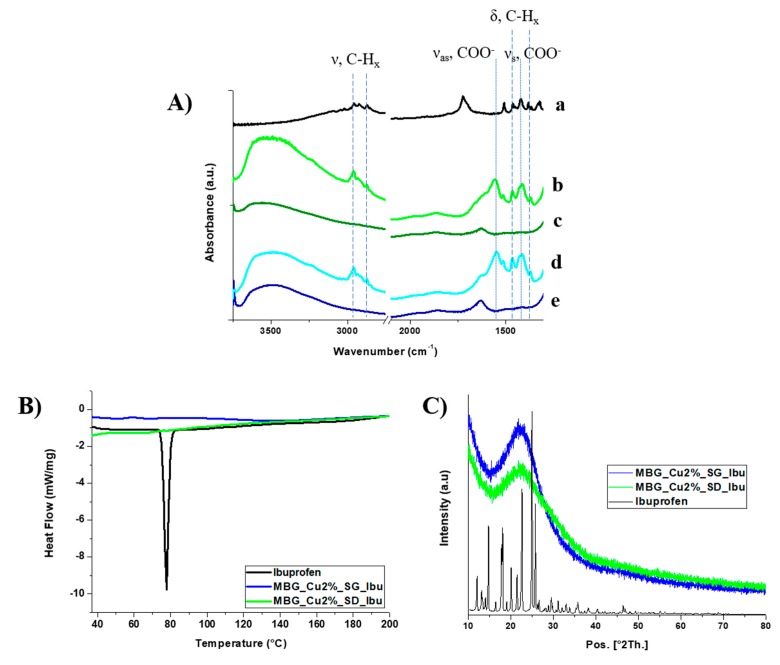
(**A**) Fourier transformed infrared (FTIR) spectra of ibuprofen (**a**), MBG_Cu 2%_SG_Ibu (**b**), MBG_Cu2%_SG (**c**), MBG_Cu 2%_SD_Ibu (**d**) and MBG_Cu2%_SD (**e**). (**B**) Differential scanning calorimetry (DSC) thermograms of ibuprofen, MBG_Cu2%_SG_Ibu and MBG_Cu2%_SD_Ibu samples. (**C**) X-ray powder diffraction (XRD) patterns of ibuprofen, MBG_Cu2%_SG_Ibu and MBG_Cu2%_SD_Ibu samples.

The FTIR spectra of Cu-substituted MBGs before (curves b–d) and after ibuprofen loading (curves c–e) are reported in Figure 3A and compared to the FTIR spectrum of ibuprofen alone (curve a). MBG samples showed the typical adsorption bands of H-bonded hydroxyls (stretching vibration) in the range of 3750–3000 cm^−1^. For what concerns drug-loaded samples, spectra showed the typical bands of ibuprofen molecule: the absorption bands at 2933 and 2871 cm^−1^ ascribed to C–H stretching modes and the signals at 1475 and 1421 cm^−1^ attributed to C–H bending vibrations. At variance with the spectrum of the drug alone which showed a clear adsorption band at 1706 cm^−1^, due to the C=O stretching vibration in –COOH groups, for ibuprofen-loaded samples two bands appeared at 1550 and 1407 cm^−1^, due to the asymmetric (ν_as_) and symmetric (ν_s_) stretching vibration of the carboxylate group COO^−^, respectively [32], resulting from proton-transfer reactions from carboxylic moieties to hydroxyl groups at MBG surface [33]. As widely reported in the literature [34,35], drug loading in their amorphous form results in an increased dissolution rates and solubility. Hence, DSC and X-ray powder diffraction (XRD) analyses of ibuprofen-loaded samples were conducted to assess the amorphous state of the drug and exclude the presence of large crystalline aggregates. Figure 3 compares the DSC thermograms of Ibu as such, MBG_Cu2%_SG_Ibu and MBG_Cu2%_SD_Ibu samples. A single endothermic melting peak at 76 °C, ascribed to crystal phase melting, was observed only for ibuprofen as such, confirming the non-crystalline state of ibuprofen loaded into the mesopores. The amorphous state of the drug was also confirmed by XRD analysis (Figure 3D). XRD pattern of ibuprofen powder showed several characteristic X-ray diffraction peaks, which completely disappeared upon drug loading into the pores of Cu-substituted MBGs, which, in accordance with DSC data, strongly suggested that re-crystallization did not occur inside the pores upon solvent evaporation during the incorporation process. This behavior has been previously reported by Bràs et al. [30], who confirmed the amorphous state of ibuprofen confined inside SBA-15 silicas with similar pore size. The proposed attribution is also supported by several authors [36,37] who reported that re-crystallization of the entrapped drug molecules is suppressed below a critical pore diameter, showing that crystallization can occur only when pore size is significantly larger (about 20 times) compared to the drug size [36].

### 3.2. Poly(ether urethane) Chemical Characterization

**Figure 4:**
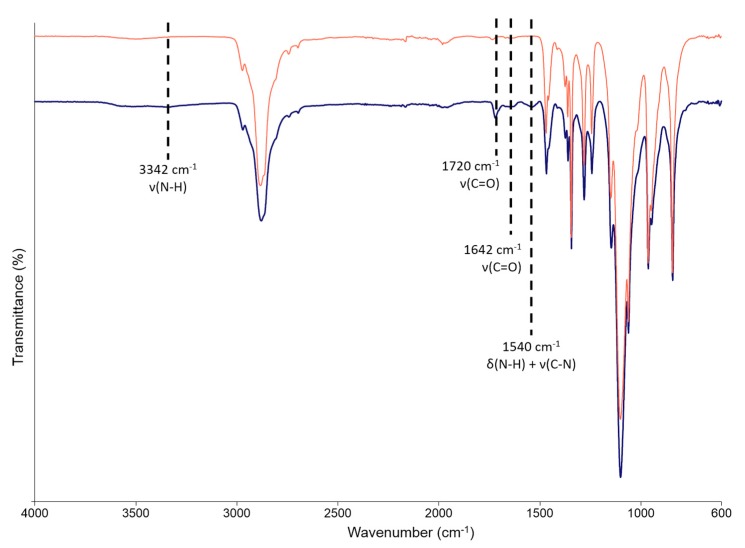
Attenuated total reflectance Fourier transformed infrared (ATR-FTIR) spectra of P407 (red) and CHP407 (blue). Dashed lines identify the characteristic signals of newly formed urethane bonds in CHP407.

The successful synthesis of a PEU carrying P407 blocks in its backbone was confirmed by attenuated total reflectance Fourier transformed infrared (ATR-FTIR) spectroscopy (Figure 4). The appearance of new transmission peaks in CHP407 spectrum, compared to P407 one, clearly proved the formation of urethane bonds among PEU building blocks [16,21]. In detail, the formation of N-H bonds was proved by the appearance of a new peak at 3342 cm^−1^ (stretching vibration), while the signals at 1720 and 1642 cm^−1^ could be ascribed to the stretching vibration of urethane free and bound carbonyl groups (C=O), respectively. N-H bonds also showed a bending vibration at 1540 cm^−1^ concurrent with C-N bond stretching. CHP407 showed M_n_ of 71,670 Da and a polydispersity index of 1.7, further confirming the success of the polymerization reaction.

### 3.3. Characterization of Hybrid Sol-Gel Systems

#### 3.3.1. Thermosensitive Behavior of CHP407-Based Hydrogels

The temperature-dependent sol-to-gel transition of the developed systems was characterized through tube inverting tests carried out in temperature ramp mode and in isothermal conditions at 37 °C. The former test allowed the estimation of hydrogel LCGT, while the latter was conducted to evaluate the time required for a complete gelation in physiological conditions. Table 3 reports LCGT values and gelation time in physiological conditions of pure CHP407 hydrogel and CHP407 hybrid hydrogels.

The incorporation of MBG_Cu2%_SG_Ibu, MBG_Cu2%_SD_Ibu or ibuprofen as such was found to slightly influence the transition kinetics of the designed sol-gel systems, in accordance with our previous work [16]. Particle incorporation marginally increased hydrogel gelation temperature, suggesting that MBG particles act as defects in the gel network, initially hindering and then slowing down the kinetics of the sol-to-gel transition [16]. On the other hand, the slight decrease of gelation time in physiological conditions observed for CHP407 gels containing MBG_Cu2%_SG_Ibu particles could also result from the criterion adopted to define the “sol” and the “gel” states, that is, presence or absence of sample flow within 30 s of vial inversion. Indeed, particle addition to the hydrogels induced an increase in viscosity that, as a consequence, inevitably accounted for the shorter incubation time at 37 °C requested for not observing any flow within the observation time. Similarly, the slightly lower gelation temperature of CHP407_MBG_Cu2%_SG_Ibu compared to CHP407_MBG_Cu2%_SD_Ibu could be correlated to their dimensional differences, which result in different hydrogel viscosity. Indeed, at a fixed MBG concentration of 20 mg/mL, the number of MBG_Cu2%_SG_Ibu contained throughout the gel is expected to be higher compared to MBG_Cu2%_SD_Ibu, due to the lower size of SG particles. Regarding the addition of ibuprofen as such, no effects were observed in gelation time in physiological conditions, while LCGT value slightly decreased. This behavior did not result from the addition of a small volume of EtOH (used to solubilize Ibu) to the sol-gel systems (data not reported), but rather to the intrinsic nature of the drug. In fact, being hydrophobic, ibuprofen is expected to be partly loaded within the core of the forming CHP407 micelles, thus inducing an increase of micelle volume, which then achieves the critical value required for the onset of thermal gelation [8] at a lower temperature. Despite the commented slight changes in LCGT and gelation time at 37 °C, neither the addition of MBG particles of different size nor the incorporation of a hydrophobic drug significantly affected the gelation potential of CHP407-based hydrogels upon temperature increase.

#### 3.3.2. Gel Characterization through Swelling and Stability to Dissolution Tests

**Figure 5:**
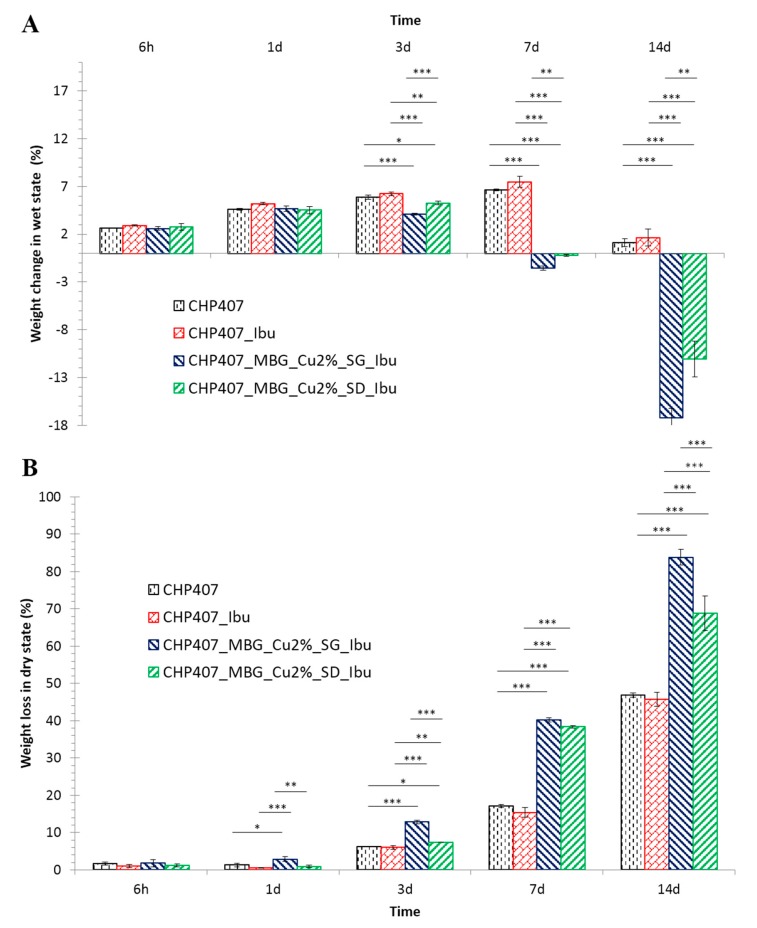
Swelling (**A**) and weight loss (**B**) of CHP407, CHP407_Ibu, CHP407_MBG_Cu2%_SG_Ibu and CHP407_MBG_Cu2%_SD_Ibu gels evaluated at 6 h, 1d, 3d, 7d and 14d.

CHP407-based gels, both virgin and hybrid formulations, were characterized in terms of aqueous medium absorption (Figure 5A) and dissolution/degradation (Figure 5B) over time in a physiological mimicking environment, that is, at 37 °C in the presence of a physiological-like buffer at pH 7.4. The incorporation of ibuprofen as such within CHP407 gels did not significantly affect gel behavior in aqueous media. Indeed, the percentage of swelling increased overtime up to 7d, followed by a drastic decrease on day 14 for both CHP407 and CHP407_Ibu samples. This change in medium absorption trend can be correlated to two concurrent phenomena occurring in the samples and their balance, namely swelling and dissolution/degradation resulting from the progressive absorption of aqueous media within the gels. The decrease in the swelling percentage observed on day 14 in CHP407 and CHP407_Ibu is thus due to the increasing gel instability in aqueous media over time. This hypothesis was demonstrated by the progressive increase in the percentage of weight loss (statistically significant increase at each time point, with the exception of day 1), that reached a value of 46.9 ± 0.6% and 45.8 ± 1.9% for CHP407 and CHP407_Ibu, respectively, after 14 days incubation in aqueous environment. Differently from ibuprofen, the embedding of MBGs in CHP407 hydrogels significantly affected gel long-term stability in aqueous environment (statistical differences between particle-loaded and not-loaded gels observed from day 3 in terms of swelling and day 1 in terms of weight loss percentage). In fact, both CHP407_MBG_Cu2%_SG_Ibu and CHP407_MBG_Cu2%_SD_Ibu showed negative swelling percentages starting from day 7 of incubation time suggesting that dissolution/degradation had completely prevailed on absorption phenomena. This observation was further proved by weight loss data, with CHP407_MBG_Cu2%_SG_Ibu and CHP407_MBG_Cu2%_SD_Ibu being almost completely dissolved/degraded after 14 days incubation in aqueous medium (weight loss percentage of 83.9 ± 2.2% and 68.9 ± 4.7% for CHP407_MBG_Cu2%_SG_Ibu and CHP407_MBG_Cu2%_SD_Ibu, respectively). The collected data confirmed the behavior previously observed for similar systems [16], with slight differences due to the different sample geometry, that is, gel thickness and extension of the surface in contact with the aqueous medium. More in detail, since day 1, CHP407_MBG_Cu2%_SG_Ibu and CHP407_MBG_Cu2%_SD_Ibu showed statistically different dissolution/degradation, while swelling percentage became significantly different from day 3 on, with hydrogel containing MBG_Cu2%_SG_Ibu exhibiting higher destabilization induced by the progressive absorption of aqueous medium.

**Figure 6:**
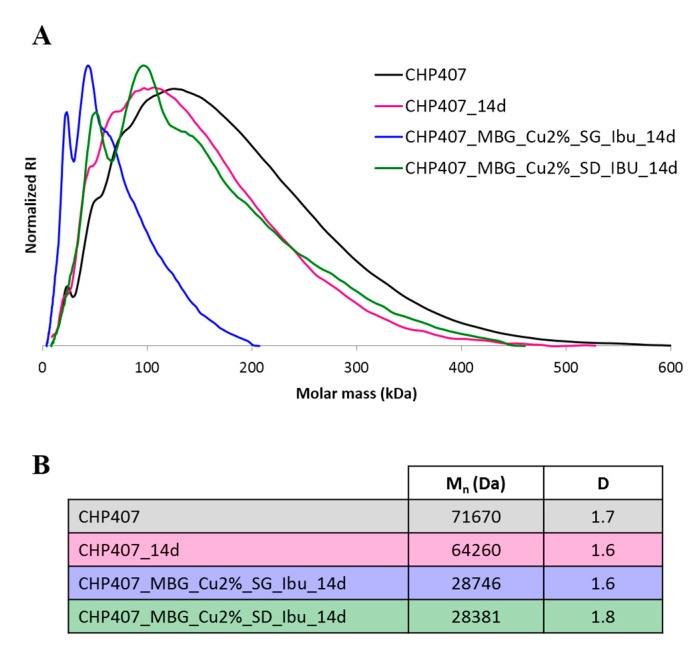
Molecular weight distribution (normalized refractive index (RI) vs. molar mass) (**A**) and estimated M_n_ and D values (**B**) of as synthesized CHP407 and residual CHP407 collected from CHP407, CHP407_MBG_Cu2%_SG_Ibu and CHP407_MBG_Cu2%_SD_Ibu gels incubated in aqueous medium for 14 days.

SEC analysis was also performed on the residual polymer phase collected on day 14 (Figure 6).

SEC revealed that the high destabilization of MBG containing gels in aqueous medium is ascribable to a progressive polymer chemical degradation, which was almost absent in pure CHP407 and CHP407_Ibu gels. In fact, CHP407 number average molecular weight decreased of about 60% and 10% in MBG-containing and pure hydrogel samples, respectively. In analogy with data concerning the bio-stability for poly(ether urethane)-based biomedical devices, such as pacemaker leads, this behavior can be ascribed to the occurrence of metal ion-mediated oxidation induced by the copper ions progressively released within the gels and the surrounding medium, which trigger the oxidative degradation of the polymer network [38,39].

#### 3.3.3. Ion/Drug Release Test

**Figure 7:**
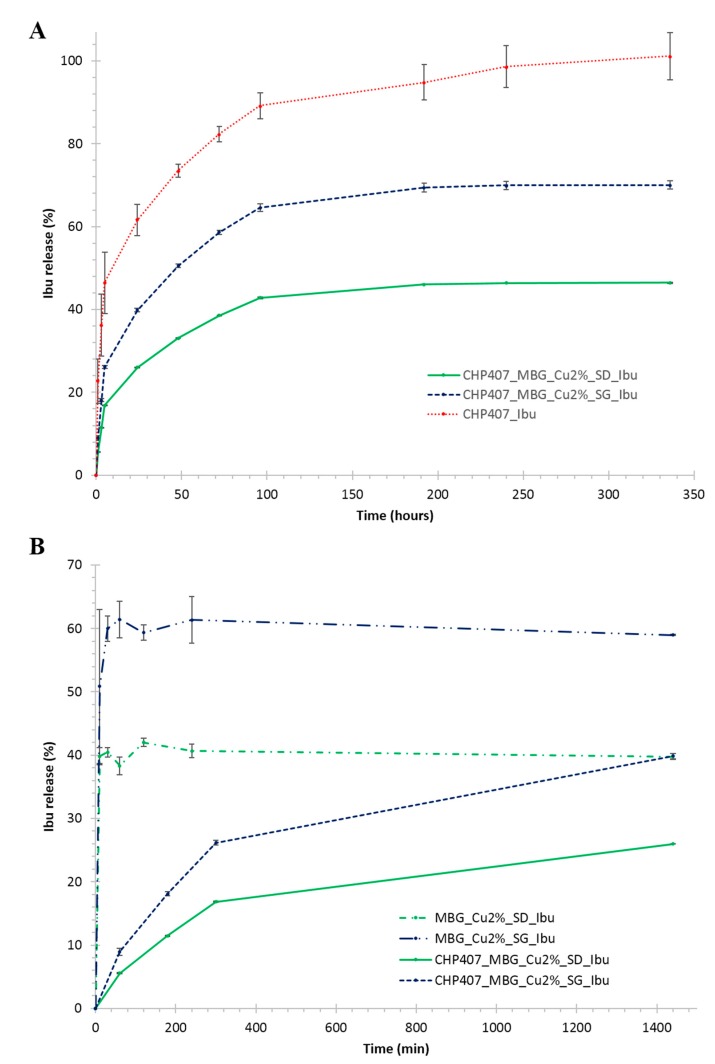
(**A**) Ibuprofen release (%) profile from CHP407_Ibu, CHP407_MBG_Cu2%_SG_Ibu and CHP407_MBG_Cu2%_SD_Ibu hydrogels. (**B**) Comparison among ibuprofen release profiles assessed from CHP407_MBG_Cu2%_SG_Ibu, CHP407_MBG_Cu2%_SD_Ibu, MBG_Cu2%_SD_Ibu and MBG_Cu2%_SD_Ibu.

Ibuprofen release profile from CHP407-based gels was assessed in physiological-like conditions, that is, Trizma^®^ buffer at 37 °C (Figure 7).

**Figure 8:**
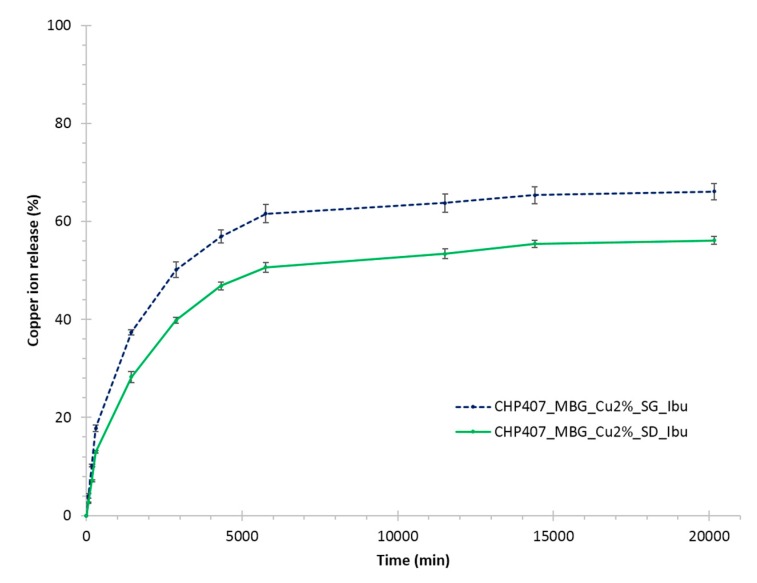
Copper ion release (%) profile from CHP407_MBG_Cu2%_SG_Ibu and CHP407_MBG_Cu2%_SD_Ibu gels.

Figure 7A compares ibuprofen release profiles from CHP407_Ibu, CHP407_MBG_Cu2%_SG_Ibu and CHP407_MBG_Cu2%_SD_Ibu up to 14 days observation time. A complete release of the drug was observed from the gels loaded with ibuprofen as such, with a release of 98.6 ± 5.0% at day 10. On the other hand, MBG-loaded gels showed a sustained and prolonged release of the drug, with a percentage release of 70.0 ± 1.0% and 46.4 ± 0.1% from CHP407_MBG_Cu2%_SG_Ibu and CHP407_MBG_Cu2%_SD_Ibu, respectively, after 14 days incubation. Moreover, starting from day 8 the release profile reached a plateau value for both particle-loaded gels, thus suggesting an incomplete ibuprofen release. However, this phenomenon cannot be avoided, being correlated to the progressive pore occlusion due to the dissolution of silica-based MBG framework and its re-precipitation as silica gel at the pores mouth, in accordance with observations reported by Mortera et al. [40], who, in addition, also reported a significant change of the delivery curves as a function of the release medium volume. To further investigate this aspect, the release profile of ibuprofen from MBG_Cu2%_SG_Ibu and MBG_Cu2%_SD_Ibu alone was studied in the same release conditions (in term of powder/medium volume ratio) adopted for hybrid hydrogel testing. As a matter of fact, both MBG_Cu2%_SG_Ibu and MBG_Cu2%_SD_Ibu showed a plateau ibuprofen release of 61.3 ± 3.7% and 40.6 ± 1.0% after 30 and 10 min incubation time, respectively. The significant difference in the maximum amount released can be attributed to the very high surface reactivity of spray-dried systems when soaked in aqueous media, leading to enhanced dissolution/re-precipitation phenomena and consequent hindered pore accessibility [16]. By comparing ibuprofen release kinetics from free MBGs and hydrogels containing MBG particles within the first 4 h, the role exerted by the polymeric matrix in modulating the release profile of ibuprofen was clearly highlighted (Figure 7B). In fact, a significant reduction (0.0001 < *p* < 0.001) in the initial burst release upon MBG incorporation within CHP407 gels was observed, with approx. 85% burst release reduction for both kinds of particles investigated. The release profile of copper ions from the developed hybrid formulations was also studied, showing a trend similar to that assessed for ibuprofen (Figure 8).

After 14 days of incubation in aqueous medium, the 66.1 ± 1.6% and 56.1 ± 0.8% (corresponding to 291.5 ± 7.1 ppm and 192.3 ± 2.7 ppm, respectively) of the copper initially present in the MBG framework was released from CHP407_MBG_Cu2%_SG_Ibu and CHP407_MBG_Cu2%_SD_Ibu, respectively. These amounts fully agree with our previous results obtained for Cu-substituted MBGs without ibuprofen [16], highlighting that drug loading within MBG porous structure did not suppress or hinder the capability to release therapeutic ions through ion-exchange reactions. The incorporation of Cu-substituted MBGs within the polymeric phase successfully decreased the undesirable initial burst release of Cu^2+^ species typically observed for particles alone (0.0001 < *p* < 0.001). Indeed, after 1h incubation in similar releasing conditions, CHP407_MBG_Cu2%_SG_Ibu and CHP407_MBG_Cu2%_SD_Ibu released an amount of copper ions approximately 72 % and 61 % lower compared to the corresponding free MBG particles. Both ibuprofen and copper ions were released faster from CHP407_MBG_Cu2%_SG_Ibu compared to CHP407_MBG_Cu2%_SD_Ibu, in accordance with the higher surface area of MBG_Cu2%_SG_Ibu compared to MBG_Cu2%_SD_Ibu, which accounts for a faster molecule diffusion, either ions or drugs, through MBG porous network.

To clarify the ion/drug release mechanism, the equation of Korsmeyer-Peppas was employed to estimate the release exponent *n* within the timeframe 1–5 h (being Korsmeyer-Peppas equation valid up to 60% release). Ibuprofen delivery from CHP407_Ibu gels turned out to be a purely Fickian diffusion-driven release, with an *n* exponent of 0.45 ± 0.04. On the other hand, CHP407_MBG_Cu2%_SG_Ibu and CHP407_MBG_Cu2%_SD_Ibu gels released ibuprofen with an anomalous mechanism (*n* value of 0.66 ± 0.03 and 0.68 ± 0.01 for CHP407_MBG_Cu2%_SG_Ibu and CHP407_MBG_Cu2%_SD_Ibu, respectively), being the drug first diffused from the MBGs within the hydrogel and then through the hydrogel in the surrounding releasing medium. Interestingly, the release exponent clearly highlighted the coexistence of diffusion and ion exchange reactions when copper species were released, being *n* values higher than 0.89 (*n* value of 0.94 ± 0.08 and 0.90 ± 0.07 for CHP407_MBG_Cu2%_SG_Ibu and CHP407_MBG_Cu2%_SD_Ibu, respectively).

Taken together the obtained data proved the ability of the developed platform to co-deliver copper ions and ibuprofen with a sustained release profile. The final released concentration can be finely modulated to target the required therapeutic effect with no associated toxicity, by taking advantage of the several degrees of freedom offered by the hybrid system, such as the initial particle concentration within the gel solution (before gelation) and the amount of ion/drug loaded inside the MBG nanocarriers.

## 4. Conclusions

In this work, a first attempt toward the design of a hybrid sol-gel formulation able to simultaneously co-release copper ions (exerting pro-angiogenic and anti-bacterial effects) and an anti-inflammatory drug (ibuprofen) was successfully achieved. To this aim, Cu-substituted MBG (2% mol) particles were loaded with ibuprofen through an incipient wetness technique, resulting in a high loading capacity, and then embedded within PEU-based thermosensitive gels at the highest possible concentration (20 mg/mL), according to an optimized incorporation protocol. Full characterization of the resulting hybrid systems was performed to highlight the effects of particle encapsulation on gelation kinetics as well as on ions/drug release mechanism. The incorporation of MBG particles within the sol-gel systems did not negatively affect their capability to undergo a temperature-driven sol-to-gel transition within a few minutes. The progressive release of Cu^2+^ species was found to play a significant role on the stability of the gels in an aqueous environment and catalyzed the oxidation of the PEU chains. The co-release of copper ions and ibuprofen from hybrid formulations was sustained and prolonged over time for up to more than one week, with a strongly reduced initial burst effect compared to MBG particles alone (2%–4% vs. 7%–14% Cu^2+^ release and 6%–9% vs. 38%–61% ibuprofen release from hybrid MBG-polyurethane formulations and free MBG particles, respectively). However, the release profile of both copper species and ibuprofen was affected by the progressive occlusion of mesopores resulting from the dissolution and the re-precipitation of silica-based MBG framework in the form of a silica gel at the pore entrance [40,41,42].

Taken together the obtained data proved the ability of the proposed hybrid thermosensitive formulation to concentrate and maintain the MBG carriers at the pathological site and to guarantee in situ and prolonged co-release of ions and drugs, thus opening the way to the design of multifunctional platforms for advanced treatment of compromised tissue healing. The high versatility of the proposed approach lies in the possibility to modulate the relative amount of the organic and inorganic components, by changing the initial particle concentration within the hydrogel solution (before gelation), and/or the initial drug loading and the chemical composition of MBGs, in order to design systems able to co-release ions and pharmaceutics with concentrations and kinetics adapted to the targeted applications and not producing cytotoxic effects.

The versatility of the herein developed hybrid sol-gel systems could thus pave the way to the treatment of a great variety of pathological conditions of soft (e.g., non-healing wounds) and hard (e.g., delayed bone healing) tissues.

## Figures and Tables

**Table 1:** Compositional information and acronyms of the designed sol-gel systems.

Acronym	Composition
CHP407	CHP407 poly(ether urethane) (PEU) at 15% *w*/*v*
CHP407_Ibu	CHP407 PEU at 15% *w*/*v* + ibuprofen at a concentration equal to the mean drug amount assessed in MBG_Cu2%_SG_Ibu and MBG_Cu2%_SD_Ibu (i.e., 2.5 mg/mL)
CHP407_MBG_Cu2%_SG_Ibu	CHP407 PEU at 15% *w*/*v* + MBG_Cu2%_SG_Ibu (20 mg/mL MBG concentration and the corresponding amount of ibuprofen)
CHP407_MBG_Cu2%_SD_Ibu	CHP407 PEU at 15% *w*/*v* + MBG_Cu2%_SD_Ibu (20 mg/mL MBG concentration and the corresponding amount of ibuprofen)

**Table 2:** Structural properties (i.e., specific surface area -SSA_BET_-, pore volume, pore size) of MBG_Cu2%_SG, MBG_Cu2%_SD, MBG_Cu2%_SG_Ibu and MBG_Cu2%_SD_Ibu.

Acronym	SSA_BET_ (m^2^ g^−1^)	Pore Volume (cm^3^ g^−1^)	Pore Size (nm)
MBG_Cu2%_SG	740	0.65	4.2
MBG_Cu2%_SG_Ibu	330	0.35	4–6
MBG_Cu2%_SD	226	0.24	8–11
MBG_Cu2%_SD_Ibu	54	0.081	8–9

**Table 3:** Lower critical gelation temperature (LGCT) and gelation time at 37 °C of CHP407, CHP407_Ibu, CHP407_ MBG_Cu2%_SG_Ibu and CHP407_ MBG_Cu2%_SD_Ibu.

Acronym	LCGT (°C) ^1^	Gelation Time @ 37 °C (min) ^2^
CHP407	28	5
CHP407_Ibu	27	5
CHP407_MBG_Cu2%_SG_Ibu	29	4
CHP407_MBG_Cu2%_SD_Ibu	30	6

^1^ Error: ± 0.5 °C. ^2^ Error: ± 0.5 min.

## Supplementary Materials

The following are available online at https://www.mdpi.com/1999-4923/11/10/501/s1, Figure S1: TEM image of MBG_Cu2%_SD.

## References

[1] Kargozar S., Montazerian M., Hamzehlou S., Kim H.W., Baino F. (2018). Mesoporous bioactive glasses: Promising platforms for antibacterial strategies. Acta Biomater..

[2] Vishnu Priya M., Sivshanmugam A., Boccaccini A.R., Goudouri O.M., Sun W., Hwang N., Deepthi S., Nair S.V., Jayakumar R. (2016). Injectable osteogenic and angiogenic nanocomposite hydrogels for irregular bone defects. Biomed. Mater..

[3] Lowe S.B., Tan V.T.G., Soeriyadi A.H., Davis T.P., Gooding J.J. (2014). Synthesis and High-Throughput Processing of Polymeric Hydrogels for 3D Cell Culture. Bioconjug. Chem..

[4] Zhu M., Zhu Y., Zhang L., Shi J. (2013). Preparation of chitosan / mesoporous silica nanoparticle composite hydrogels for sustained co-delivery of biomacromolecules and small chemical drugs. Sci. Technol. Adv. Mater..

[5] Zhao P., Liu H., Deng H., Xiao L., Qin C. (2014). Colloids and Surfaces B: Biointerfaces A study of chitosan hydrogel with embedded mesoporous silica nanoparticles loaded by ibuprofen as a dual stimuli-responsive drug release system for surface coating of titanium implants. Colloids Surf. B Biointerfaces.

[6] Lunter D.J. (2017). Evaluation of mesoporous silica particles as drug carriers in hydrogels. Pharm. Dev. Technol..

[7] Kim B.S., Chen Y., Srinoi P., Marquez M.D., Lee T.R. (2019). Hydrogel-Encapsulated Mesoporous Silica-Coated Gold Nanoshells for Smart Drug Delivery. Int. J. Mol. Sci..

[8] Lei L., Liu Z., Pingyun Y., Yuan P., Jin R., Wang X.-d., Jiang T., Chen X. (2019). Injectable colloidal hydrogel with mesoporous silica nanoparticles for sustained co-release of microRNA-222 and aspirin to achieve innervated bone regeneration in rat mandibular defects. J. Mater. Chem. B.

[9] Fiorilli S., Molino G., Pontremoli C., Iviglia G., Torre E., Cassinelli C., Morra M., Vitale-Brovarone C. (2018). The incorporation of strontium to improve bone-regeneration ability of mesoporous bioactive glasses. Materials.

[10] Zhou Y., Han S., Xiao L., Han P., Wang S., He J., Chang J., Wu C., Xiao Y. (2018). Accelerated host angiogenesis and immune responses by ion release from mesoporous bioactive glass. J. Mater. Chem. B.

[11] Wu C., Zhou Y., Xu M., Han P., Chen L., Chang J., Xiao Y. (2013). Copper-containing mesoporous bioactive glass scaffolds with multifunctional properties of angiogenesis capacity, osteostimulation and antibacterial activity. Biomaterials.

[12] Wang X., Cheng F., Liu J., Smått J.H., Gepperth D., Lastusaari M., Xu C., Hupa L. (2016). Biocomposites of copper-containing mesoporous bioactive glass and nanofibrillated cellulose: Biocompatibility and angiogenic promotion in chronic wound healing application. Acta Biomater..

[13] Bari A., Bloise N., Fiorilli S., Novajra G., Vallet-Regí M., Bruni G., Torres-Pardo A., González-Calbet J.M., Visai L., Vitale-Brovarone C. (2017). Copper-containing mesoporous bioactive glass nanoparticles as multifunctional agent for bone regeneration. Acta Biomater..

[14] Li H., Li J., Jiang J., Lv F., Chang J., Chen S., Wu C. (2017). An osteogenesis / angiogenesis-stimulation artificial ligament for anterior cruciate ligament reconstruction. Acta Biomater..

[15] Romero-sánchez L.B., Marí-beffa M., Carrillo P., Ángel M., Díaz-cuenca A. (2018). Copper-containing mesoporous bioactive glass promotes angiogenesis in an in vivo zebrafish model. Acta Biomater..

[16] Pontremoli C., Boffito M., Fiorilli S., Laurano R., Torchio A., Bari A., Tonda-Turo C., Ciardelli G., Vitale-Brovarone C. (2018). Hybrid injectable platforms for the in situ delivery of therapeutic ions from mesoporous glasses. Chem. Eng. J..

[17] Vergara-figueroa J., Alejandro-mart S., Pesenti H. (2019). Obtaining Nanoparticles of Chilean Natural Zeolite and its Ion Exchange with Copper Salt (Cu^2+^) for antibacterial applications. Materials (Basel).

[18] Rosenberg M., Vija H., Kahru A., Keevil C.W., Ivask A. (2018). Rapid in situ assessment of Cu-ion mediated effects and antibacterial efficacy of copper surfaces. Sci. Rep..

[19] Barralet J., Gbureck U., Habibovic P., Vorndran E., Gerard C., Doillon C.J. (2009). Angiogenesis in Calcium Phosphate Scaffolds by Inorganic Copper Ion Release. Tissue Eng. Part A.

[20] Charnay C., Bégu S., Tourné-Péteilh C., Nicole L., Lerner D.A., Devoisselle J.M. (2004). Inclusion of ibuprofen in mesoporous templated silica: Drug loading and release property. Eur. J. Pharm. Biopharm..

[21] Boffito M., Gioffredi E., Chiono V., Calzone S., Ranzato E., Martinotti S., Ciardelli G. (2016). Novel polyurethane-based thermosensitive hydrogels as drug release and tissue engineering platforms: Design and in vitro characterization. Polym. Int..

[22] Alsirawan M.B., Mohammad M.A., Alkasmi B., Alhareth K., El-Hammadi M. (2013). Development and validation of a simple HPLC method for the determination of ibuprofen sticking onto punch faces. Int. J. Pharm. Pharm. Sci..

[23] Boffito M., Brancot A.G., Lima O., Bronco S., Sartori S., Ciardelli G., Torino P. (2019). Injectable thermosensitive gels for the localized and controlled delivery of biomolecules in tissue engineering/regenerative medicine. Biomed. Sci. Eng..

[24] El-Fiqi A., Kim T.-H., Kim M., Eltohamy M., Won J.-E., Lee E.-J., Kim H.-W. (2012). Capacity of mesoporous bioactive glass nanoparticles to deliver therapeutic molecules. Nanoscale.

[25] Vallet-Regí M. (2006). Ordered mesoporous materials in the context of drug delivery systems and bone tissue engineering. Chem. Eur. J..

[26] Mellaerts R., Jammaer J.A., Van Speybroeck M., Chen H., Van Humbeeck J., Augustijns P., Van den Mooter G., Martens J.A. (2008). Physical state of poorly water soluble therapeutic\rmolecules loaded into SBA-15 ordered mesoporous silica carriers:\rA case study with itraconazole and ibuprofen. Langmuir.

[27] Gonzalez G., Sagarzazu A., Zoltan T. (2013). Infuence of Microstructure in Drug Release Behavior of Silica Nanocapsules. J. Drug Deliv..

[28] Hong S., Shen S., Tan D.C.T., Ng W.K., Liu X., Chia L.S.O., Irwan A.W., Tan R., Nowak S.A., Marsh K. (2016). High drug load, stable, manufacturable and bioavailable fenofibrate formulations in mesoporous silica: A comparison of spray drying versus solvent impregnation methods. Drug Deliv..

[29] López-Noriega A., Arcos D., Izquierdo-Barba I., Sakamoto Y., Terasaki O., Vallet-Regí M. (2006). Ordered mesoporous bioactive glasses for bone tissue regeneration. Chem. Mater..

[30] Brás A.R., Merino E.G., Neves P.D., Fonseca I.M., Dionísio M., Schönhals A., Correia N.T. (2011). Amorphous ibuprofen confined in nanostructured silica materials: A dynamical approach. J. Phys. Chem. C.

[31] Zhang Y., Zhi Z., Jiang T., Zhang J., Wang Z., Wang S. (2010). Spherical mesoporous silica nanoparticles for loading and release of the poorly water-soluble drug telmisartan. J. Control. Release.

[32] Fiorilli S., Onida B., Bonelli B., Garrone E. (2005). In situ infrared study of SBA-15 functionalized with carboxylic groups incorporated by a Co-condensation route. J. Phys. Chem. B.

[33] Cauda V., Fiorilli S., Onida B., Vernè E., Vitale Brovarone C., Viterbo D., Croce G., Milanesio M., Garrone E. (2008). SBA-15 ordered mesoporous silica inside a bioactive glass-ceramic scaffold for local drug delivery. J. Mater. Sci. Mater. Med..

[34] Wang F., Hui H., Barnes T.J., Barnett C., Prestidge C.A. (2010). Oxidized mesoporous silicon microparticles for improved oral delivery of poorly soluble drugs. Mol. Pharm..

[35] Shen S.C., Ng W.K., Hu J., Letchmanan K., Ng J., Tan R.B.H. (2017). Solvent-free direct formulation of poorly-soluble drugs to amorphous solid dispersion via melt-absorption. Adv. Powder Technol..

[36] Sliwinska-Bartkowiak M., Dudziak G., Gras R., Sikorski R., Radhakrishnan R., Gubbins K.E. (2001). Freezing behavior in porous glasses and MCM-41. Colloids Surf. A Physicochem. Eng. Asp..

[37] Radhakrishnan R., Gubbins K.E., Sliwinska-Bartkowiak M. (2000). Effect of the fluid-wall interaction on freezing of confined fluids: Toward the development of a global phase diagram. J. Chem. Phys..

[38] Thoma R.J., Tan F.R., Phillips R.E. (1988). Ionic Interactions of Polyurethanes. J. Biomater. Appl..

[39] Coury A.J. (2013). Chemical and Biochemical Degradation of Polymers Intended to be Biostable. Biomaterials Science: An Introduction to Materials in Medicine.

[40] Mortera R., Fiorilli S., Garrone E., Verné E., Onida B. (2010). Pores occlusion in MCM-41 spheres immersed in SBF and the effect on ibuprofen delivery kinetics: A quantitative model. Chem. Eng. J..

[41] Li P., Nakanishi K., Kokubo T., de Groot K. (1993). Induction and morphology of hydroxyapatite, precipitated from metastable simulated body fluids on sol-gel prepared silica. Biomaterials.

[42] Li P., Kangasniemi I., de Groot K., Kokubo T., Yli-Urpo A.U. (1994). Apatite crystallization from metastable calcium phosphate solution on sol-gel-prepared silica. J. Non-Cryst. Solids.

