# Supplementary material for: Injectable Thermosensitive Formulation Based on Polyurethane Hydrogel/Mesoporous Glasses for Sustained Co-Delivery of Functional Ions and Drugs
Source: Pharmaceutics. 2019 Oct 1;11(10):501. doi: 10.3390/pharmaceutics11100501 (PMC6835912; doi:10.3390/pharmaceutics11100501)
Supplement: Supplementary file 1 [file pharmaceutics-11-00501-s001.pdf]

pharmaceutics

Supplementary Materials: Injectable Thermosensitive Formulation Based on Polyurethane Hydrogel/Mesoporous Glasses for Sustained Co-Delivery of Functional Ions and Drugs

Monica Boffito, Carlotta Pontremoli, Sonia Fiorilli, Rossella Laurano, Gianluca Ciardelli and Chiara Vitale-Brovarone

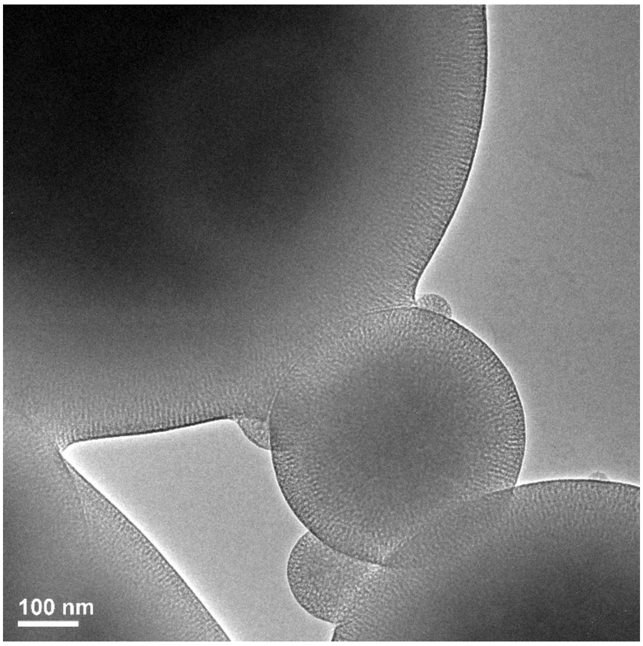

Figure S1. TEM image of MBG_Cu2%_SD.
